# HyAR-PPO: Hybrid Action Representation Learning for Incentive-Driven Task Offloading in Vehicular Edge Computing

**DOI:** 10.3390/s26061743

**Published:** 2026-03-10

**Authors:** Wentao Wang, Mingmeng Li, Honghai Wu

**Affiliations:** 1 The School of Information Engineering, Henan University of Science and Technology, Luoyang 471023, China; wentaowang2026@126.com; 2 School of Computer and Artificial Intelligence, Zhengzhou University, Zhengzhou 450001, China; lmm2090279451@stu.zzu.edu.cn

**Keywords:** vehicular edge computing, task offloading, deep reinforcement learning, hybrid action space, Nash bargaining

## Abstract

Vehicular Edge Computing (VEC) can effectively guarantee the service experience of user vehicles, but resource-limited Roadside Units (RSUs) may face insufficient computing capacity during task peak periods. Utilizing Assisting Vehicles (AVs) with idle resources to share computing power can alleviate the pressure on RSUs. However, existing studies often fail to adequately incentivize selfish assisting vehicles to contribute resources and frequently lack a global optimization perspective from the overall system welfare. To address these challenges, this paper proposes an incentive-driven utility-balanced task offloading framework that aims to maximize social welfare while jointly optimizing resource allocation and profit pricing. Specifically, we first formulate the resource allocation as a Mixed-Integer Nonlinear Programming (MINLP) problem. To solve this problem, we introduce hybrid action representation learning to VEC for the first time and propose the HyAR-PPO algorithm to jointly optimize discrete offloading decisions and continuous resource allocation. This algorithm maps heterogeneous hybrid actions to a unified latent representation space through a Variational Autoencoder for the solution. Subsequently, equilibrium prices among user vehicles, Computation Service Providers (CSPs), and assisting vehicles are determined through Nash bargaining games, satisfying individual rationality constraints and achieving Pareto-optimal fair profit distribution. Experimental results demonstrate that the proposed framework can effectively coordinate multi-party interests. Compared with mainstream methods, the approach based on hybrid action representation learning achieves a significant improvement in social welfare, with its advantages being more pronounced in medium-to-large-scale scenarios.

## 1. Introduction

In recent years, with the rise of Internet of Things (IoT) technology, the concept of smart cities is gradually transitioning from theory to reality. As an important component of smart cities, intelligent vehicles play a key role in providing smart services such as in-vehicle multimedia, driving safety, vehicle augmented reality, and autonomous driving [1,2,3,4]. Although in-vehicle CPUs are developing rapidly, they still face challenges in processing large numbers of complex applications in a short time. Furthermore, considering constraints such as current physical architecture, communication environment, and infrastructure costs, offloading tasks to remote clouds causes communication delays of 30 ms to 100 ms [5,6], which cannot meet the latency requirements of intelligent vehicle services. However, it should be noted that not all vehicular applications demand such stringent latency; many practical tasks, such as vehicular augmented reality, multimedia processing, and edge data analytics, have delay tolerances on the order of seconds [7]. In addition, the secure deployment of vehicular networks relies on robust authentication and privacy-preserving mechanisms [8,9,10], which provide the trust foundation for the task offloading interactions discussed in this paper.

Fortunately, Vehicular Edge Computing (VEC) has emerged as a promising task processing technology that can significantly reduce latency and transmission costs by deploying edge nodes with computing resources (such as RSUs) near vehicles [11,12]. However, compared to cloud centers, the limited computing resources of RSUs make it difficult to guarantee the Quality of Service (QoS) for User Vehicles (UVs) during peak task request periods [13]. A straightforward approach is to deploy more RSUs, but this inevitably leads to resource waste during off-peak periods.

To address the above issues, renting idle computing resources from Assisting Vehicles (AVs) is a better solution, which can not only alleviate the service pressure on VEC but also further reduce communication latency [14]. However, this new solution also introduces new challenges that need to be addressed: (1) introducing AVs increases the dimensionality of the policy space, making the optimization problem more complex, especially since offloading decisions involve hybrid action spaces containing both discrete variables (offloading location selection) and continuous variables (resource allocation ratio); (2) RSUs and AVs with computing resources lack reasonable motivation to participate in task offloading, i.e., they will not provide services to UVs without any compensation [15]; (3) existing studies often use Stackelberg games for pricing, which faces fairness challenges in practice due to its inherent asymmetric decision structure under information asymmetry; in contrast, mechanisms such as bargaining games promote fair allocation through negotiation [16,17].

To address the above challenges, this paper proposes an incentive-driven utility-balanced task offloading framework. This framework first models the multi-party interaction as a mixed-integer nonlinear programming problem with social welfare maximization as the objective; then, to effectively solve this NP-hard problem, we propose the HyAR-PPO algorithm based on hybrid action representation, which learns latent action representations through variational autoencoders to achieve end-to-end optimization; finally, we introduce a Nash bargaining-based pricing strategy that incentivizes assisting vehicles to participate while ensuring fairness in profit distribution.

The main contributions of this paper are summarized as follows:

1. We propose an incentive-driven utility-balanced offloading framework with social welfare maximization for task offloading decisions involving multiple self-interested entities in VEC. The framework includes a set of user vehicles, a CSP managing RSUs, and a set of assisting vehicles, where user vehicles need to make optimal decisions among abandoning tasks, local execution, offloading to RSUs, or assisting vehicles. We construct differentiated utility functions for each entity based on their unique roles, emphasizing their specific functions in the framework.
2. We propose the HyAR-PPO algorithm based on hybrid action representation to solve the social welfare maximization problem. This algorithm uses a learnable embedding table to map discrete actions into continuous vector representations and a conditional Variational Autoencoder (VAE) to encode continuous parameters into a latent space conditioned on the state and discrete action embedding, enabling the policy network to learn decisions in a unified, semantically rich representation space.
3. We design Nash bargaining-based pricing strategies to incentivize all roles to fully participate in the offloading system, where payments between user vehicles and CSP, and between CSP and assisting vehicles satisfy Individual Rationality (IR) and achieve Pareto-optimal solutions.
4. We conduct extensive comparative experiments to validate the effectiveness of the proposed methods. Results show that the hybrid action representation learning-based approach can effectively coordinate multi-party interests, achieving an average social welfare improvement of approximately 11% compared with mainstream methods, with more pronounced advantages in medium-to-large scale scenarios.

The remainder of this paper is organized as follows: Section 2 reviews related work; Section 3 describes the system architecture and establishes the problem model; Section 4 elaborates on the solution method for social welfare maximization; Section 5 discusses the Nash bargaining-based pricing strategy; Section 6 analyzes experimental results; Section 7 concludes the paper and outlines future work.

## 2. Related Work

How to incentivize vehicles or devices to contribute computing resources and price computing resources are current research hotspots, with methods based on game theory, auction theory, and machine learning being widely applied. In this section, we first discuss research progress on incentive mechanisms and resource pricing in edge computing networks, then discuss research on reinforcement learning applications in edge computing and hybrid action reinforcement learning.

### 2.1. Incentive Mechanism Research in Edge Computing

In edge computing networks, resource providers aim to maximize their own profits while end users expect to obtain computing resources at minimum cost, creating an inherent conflict of interest between the two parties. To coordinate this conflict, researchers have proposed various incentive mechanisms, including game theory, auction theory, and contract theory. Among these, resource pricing, as the most direct incentive approach, balances the interests of both supply and demand sides through reasonable price setting, and has become an important research direction in this field.

Tong et al. [17] propose and comprehensively analyze three dynamic pricing mechanisms: auction-based proportional resource allocation based on non-cooperative games, and uniform pricing and differentiated pricing mechanisms based on Stackelberg games. Although this work proposes multiple pricing base models, it ignores the impact of task attribute factors on pricing. Baek et al. [18] introduce Stackelberg games to characterize the relationship between edge servers and users, proposing both uniform pricing and differentiated pricing algorithms that improve server revenue while effectively reducing user task latency. Habiba et al. [19] design an offloading service market based on repeated auctions, considering dynamically arriving offloading requests and real-time computing loads of servers and propose a computationally efficient improved generalized second-price algorithm. Cheng et al. [20] consider the impact of user task allocation strategies on edge revenue, proposing two pricing and resource allocation schemes with different granularities based on Stackelberg games. Zhao et al. [21] introduce a novel multi-round Stackelberg game framework that combines the hierarchical structure of container images to enhance resource management in Multi-access Edge Computing (MEC) networks. Ren et al. [22] propose a trustworthy dependent task offloading framework, designing centralized and distributed auction mechanisms.

In terms of incentive mechanisms, contract theory, auction theory, and game theory have been widely applied to task offloading and resource allocation in edge computing networks. Wang et al. [23] model the competition and cooperation relationships between vehicles as a two-stage Stackelberg game, focusing on vehicle social attributes and their impact on offloading decisions, proposing a fuzzy logic-based dynamic pricing strategy. Kazmi et al. [24] propose a contract theory-based incentive mechanism that provides customized contracts for each resource-sharing vehicle to provide appropriate rewards. Ma et al. [25] propose a dual-incentive mechanism based on reputation and contracts, using graph neural networks to evaluate node reputation and employing blockchain to store and manage node reputation. Zhang et al. [26] propose an incentive mechanism based on satellite–ground integrated vehicular networks, incorporating auction theory into mechanism design. Cui et al. [27] study the interaction behavior of both parties using a Stackelberg game approach in delay-tolerant crowdsensing scenarios. Liu et al. [28] propose a loss aversion-based incentive mechanism, designing incentive thresholds and threshold factors to stimulate vehicle node cooperation. Li et al. [29] establish a reward-driven incentive mechanism for mobile AIGC services in VEC, designing a multi-stage Stackelberg game under information asymmetry and employing PPO for pricing optimization. Liu et al. [30] propose a reputation-based incentive mechanism for UAV-assisted edge computing, addressing data credibility and device selfishness in the offloading process. Fan et al. [31] propose a joint trajectory scheduling and incentive mechanism for spatio-temporal urban vehicular crowd sensing, employing reverse combinatorial auctions to incentivize vehicle participation under uncertain future trajectory information; unlike their sensing-coverage objective and auction-based design, our work focuses on computation offloading resource allocation and adopts Nash bargaining to achieve Pareto-optimal multi-party profit distribution.

Regarding multi-type computing resource collaboration, many studies innovatively design frameworks that utilize multiple computing resources for collaboration to improve resource utilization and service provider utility. Wang et al. [32] use Non-Orthogonal Multiple Access (NOMA) technology to simultaneously offload computing tasks to RSUs and vehicle platoons, constructing a game model, including ISAC vehicles, RSUs, and platoons based on Stackelberg games. Song et al. [33] design resource management and pricing mechanisms to quantify the benefits and costs of all parties, constructing multi-party cooperative task offloading modes through two-stage Stackelberg. Zhao et al. [34] propose a contract-based framework to handle joint task offloading, resource sharing, and computing incentives under information asymmetry. Xue et al. [14] formulate a Stackelberg game between user vehicles and service providers, proposing a reverse auction-based task scheduling algorithm to select assisting computing vehicles. Chen et al. [35] propose a two-tier bargaining incentive mechanism for task offloading and collaborative computing. Li et al. [16] propose an incentive-based multi-level task allocation framework, using bargaining games to determine initial offloading decisions and fee payments, and employing double auction mechanisms to incentivize assisting devices to participate in task processing.

Unlike the above articles, this paper considers the problem from the perspective of resource-contributing groups’ interests, focusing on scenarios where a single MEC server actively recruits collaborative vehicles due to resource constraints. We consider user task value characteristics, combining cost and value to simulate user offloading decision behavior in edge computing networks, characterizing user offloading decisions from cost and value perspectives, and systematically evaluating the impact of incentive strategies on the overall edge ecosystem, focusing on reasonable pricing of computing resources and balanced incentive distribution.

### 2.2. Reinforcement Learning Applications in Edge Computing

In recent years, deep reinforcement learning (DRL) has been widely applied to task offloading and resource allocation problems in edge computing networks due to its adaptive decision-making capability in dynamic environments. Nu Kyi and Si Mar Win [36] provide a comprehensive review of RL-based task scheduling approaches in edge computing, highlighting the potential of RL techniques, including Q-learning, deep Q-learning, and actor–critic models. Zhao et al. [15] propose a deep reinforcement learning-based contract incentive mechanism, establishing a joint task offloading and resource cooperation optimization problem. Meng et al. [4] propose a contract-incentive partial task offloading framework, using deep reinforcement learning to design task offloading and resource allocation algorithms. Zhang et al. [37] develop a mobility-aware model that uses a deep reinforcement learning-assisted double auction model to effectively allocate base station resources. Tong et al. [38] employ multi-agent deep deterministic policy gradient algorithms to achieve coordinated multi-base station and multi-user strategies in multi-base station collaborative mobile edge environments. Ma et al. [39] apply deep reinforcement learning to vehicular cloud-assisted mobile edge computing systems, proposing a queue stability-based computation offloading scheme to balance task latency and system stability. At a broader edge-computing level, Xu et al. [40] address heterogeneous task management through task-grained federated continual learning to overcome catastrophic forgetting across non-stationary data distributions, while Luo et al. [41] analyze cost-effective adaptive federated learning in mobile edge networks by jointly optimizing client selection and local training iterations to minimize total cost under convergence constraints; both works underscore the importance of efficient resource management at the edge. The action space types of the above methods are relatively homogeneous, while actual task offloading scenarios often involve both discrete variables (offloading location selection) and continuous variables (resource allocation ratio) simultaneously, forming hybrid action spaces, which pose additional challenges for DRL algorithm design.

Therefore, in recent years, an increasing number of studies directly adopt hybrid action space DRL methods to jointly solve offloading and resource allocation problems in VEC/MEC. Shang et al. [42] propose the H2AC method, which simultaneously obtains server selection (discrete actions) and offloading rates (continuous variables) in NOMA-MEC systems to minimize computation latency and energy consumption. Yang et al. [43] adopt a hybrid average reward proximal policy optimization algorithm, jointly optimizing offloading decisions, offloading transmission rates, and edge server allocation to minimize long-term average task execution latency. Hu et al. [44] combine D3QN and DDPG networks to determine server selection, offloading rates, power, and other hybrid variables in edge–cloud collaborative networks to minimize long-term costs of MEC servers. Wang et al. [45] employ parameterized deep Q-network (PDQN) algorithms for solving users’ offloading and resource allocation hybrid action problems in multi-MEC server scenarios. Fan et al. [46] propose H-PPO (Hybrid Proximal Policy Optimization), which adopts a hybrid Actor–Critic architecture that uses multiple parallel sub-networks to separately handle discrete action policies and continuous parameter policies, sharing a single Critic network for policy optimization. Based on H-PPO, Meng et al. [4] propose the HORA algorithm for VEC task offloading, directly solving the social welfare maximization problem in the hybrid action space. However, these methods still operate directly in the original action space without learning compact representations to strengthen the correlation between discrete actions and continuous parameters.

The original HyAR framework [47] innovatively introduces the idea of learning latent action representations through variational autoencoders, providing a novel representation learning perspective for handling hybrid action space problems, and demonstrating its potential in improving sample efficiency and generalization ability on robotic control tasks. However, applying HyAR to VEC requires two key adaptations: (1) an action masking mechanism to ensure decoded actions satisfy the coupled constraints of the MINLP problem; and (2) a stochastic policy design to integrate HyAR with the on-policy algorithm PPO, replacing the original deterministic TD3 backbone. Inspired by this, our work systematically introduces the idea of hybrid action representation learning into the VEC domain for the first time. Our main contribution lies in designing a complete incentive-driven task offloading framework for the unique challenges in VEC scenarios, such as resource constraints and multi-entity utility coupling. In this framework, we first adapt the representation learning paradigm into the HyAR-PPO algorithm to solve the core resource allocation problem (MINLP); then, we integrate it into the overall mechanism of task offloading and resource pricing driven by Nash bargaining, thereby jointly achieving the overall goals of system utility maximization and fair distribution of multi-party interests.

## 3. System Model and Problem Formulation

In this section, we first describe the system scenario and explain the entire business process. Then, we provide detailed descriptions of the sub-modules in the system. Finally, we give the utility functions of participating entities and establish the optimization problem.

### 3.1. System Architecture

**Figure 1:**
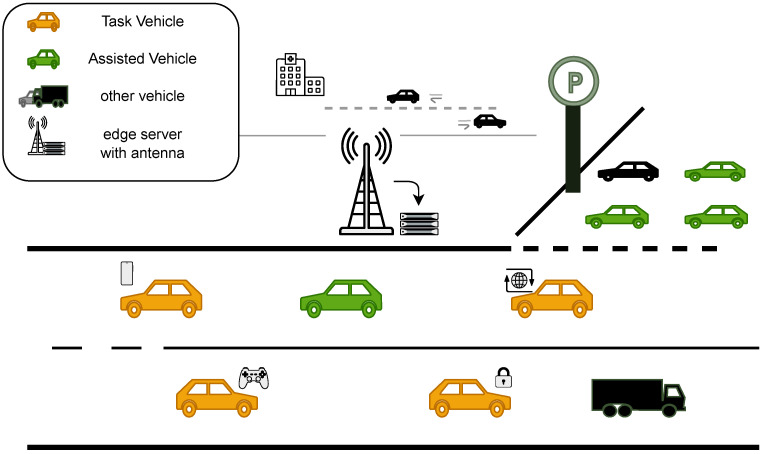
System scenario.

As shown in Figure 1, in the VEC scenario, computation-intensive tasks generated by task vehicles have high requirements for latency and computing resources, but their local computing capacity is limited and cannot complete task processing. Therefore, task vehicles need to pay certain fees to offload tasks to the server. However, if the service provider as a resource provider prices too high, task vehicles may abandon offloading due to excessive costs and directly discard tasks. At the same time, due to limited computing resources of the server, it is difficult to process too many tasks simultaneously, causing potential economic losses. We observe that there are some vehicles in the system with abundant computing resources that are in an idle state. The CSP can recruit these vehicles as assisting vehicles for task computation during resource overload, thereby obtaining more profits. However, assisting vehicles as third-party resource providers are selfish and will only provide computing services when they receive sufficient incentives without harming their own interests. This potential conflict of interest among entities requires the CSP to design reasonable pricing and incentive mechanisms to promote multi-party collaboration and optimize resource allocation.

**Figure 2:**
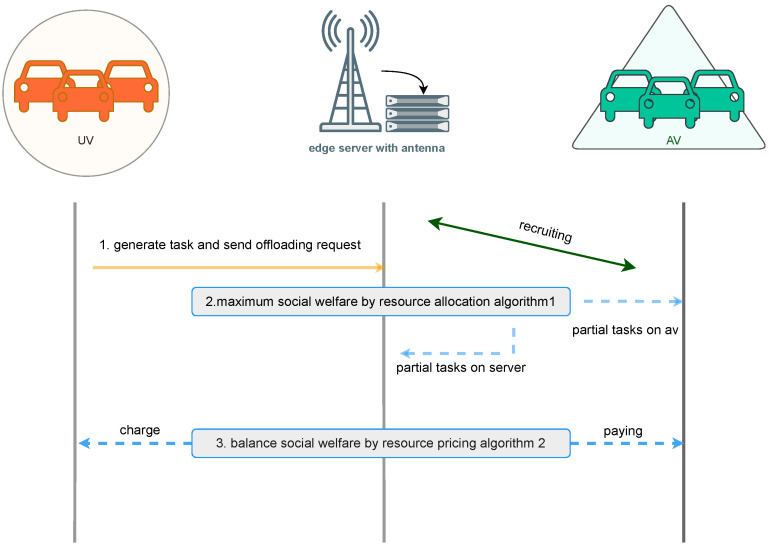
Business workflow.

As shown in Figure 2, the workflow diagram describes the business process in this scenario in detail:

- **Process 1:** There are a set of task vehicles I={1,2,…,I}, each generating a task $T_{i}=\left\{L_{i},C_{i},t_{i}^{\max},V_{i},\lambda_{i}\right\}$ uploaded to the server for execution. Here, $L_{i}$ represents the data size of the task, $C_{i}$ represents the CPU cycles required per unit data of the task, $t_{i}^{\max}$ represents the maximum tolerable delay of the task, $V_{i}$ represents the user’s task valuation, and $\lambda_{i}$ represents the value decay factor of the task.
- **Process 2:** The server has limited computing resources F and accepts task execution requests submitted by users. It executes the resource allocation Algorithm 1 and maximizes social welfare. If computing resources cannot meet all task computing requirements, some overloaded tasks are outsourced to assisting vehicles J={1,2,…,J}. Some tasks are executed on the local server with allocated resources $f_{i}^{server}$. Others are outsourced to assisting vehicles, which have computing resources $f_{j}$ and provide computing resources $f_{i}^{j}$.
- **Process 3:** Since computation consumes energy and assisting vehicles are selfish, they will only provide computing resources for tasks when they receive sufficient compensation without harming their own interests. On the other hand, if the CSP charges users too high fees, task users will choose to abandon task offloading. Therefore, the CSP executes the bargaining-based three-party pricing Algorithm 2 to balance the benefits of all parties.

For convenience of reference, Table 1 summarizes the symbols used in this paper.

### 3.2. Communication Model

This paper sets the Vehicle-to-RSU (V2R) communication model based on NOMA technology [7,32], where the transmission power for vehicle–server communication is determined by the server. The maximum transmission power of the server is set as $\theta_{server}$, and the transmission power of vehicles is $\theta_{allowed}=\frac{\theta_{server}}{\left|K\right|}$. Here, *K* represents the number of vehicles participating in task offloading during the current time period, including task vehicles and assisting vehicles, i.e., |K|=|I|+|J|. The channel gain is set as $h_{k}=\frac{\eta }{dist_{k}^{\phi /2}}$, where k∈K represents any participating vehicle, $dist_{k}$ represents the distance between vehicle *k* and the server, η is the small-scale fading following Rayleigh distribution with η∼CN(0,1), and ϕ is the large-scale path loss coefficient.

In the framework of this paper, the communication SINR between any vehicle *k* (including task vehicles and assisting vehicles) and the server can be expressed as:

(1) $$SINR_{k}=\frac{|h_{k}{|}^{2}\theta_{allowed}}{I_{k}+N_{0}}$$

where $I_{k}$ is the transmission interference. Since this scenario only has a single server, inter-edge interference is not considered. Therefore, the interference term only includes intra-edge interference, and $N_{0}$ is a constant representing Gaussian white noise. $I_{k}=\sum_{k^{\prime }\in K\setminus \left\{k\right\}}{\left|h_{k^{\prime }}\right|}^{2}\theta_{allowed}$, representing the communication interference from other vehicles within the edge to vehicle *k*, where $k^{\prime }$ satisfies $\left\{k^{\prime }||h_{k^{\prime }}{|}^{2}<|h_{k}{|}^{2},\forall k^{\prime }\in K\setminus \left\{k\right\}\right\}$. Therefore, the transmission rate between vehicle *k* and the server can be expressed as:

(2) $$R_{k}=B\log_{2}\left(1+SINR_{k}\right)$$

### 3.3. Computation Model

The task computation offloading process consists of task transmission and task execution. Since the data size of execution results is much smaller than the input data size, the return time of task execution results is generally negligible [14]. Below, the unit energy price γ is used to convert energy consumption at each stage directly into monetary costs.

(1) Task Transmission: The transmission time for task vehicle *i* to upload a task to the server is:

(3) $$t_{i,server}^{tran}=\frac{L_{i}}{R_{i}}$$

The transmission cost is:

(4) $$M_{i,server}^{tran}=\gamma \theta_{i}^{allowed}\frac{L_{i}}{R_{i}}$$

where $R_{i}$ is the transmission rate between task vehicle *i* and the server. Similarly, the transmission time and cost when the server forwards a task to assisting vehicle *j* are denoted as $t_{(i,server),j}^{tran}$ and $M_{(i,server),j}^{tran}$, respectively, obtained by replacing $R_{i}$ and $\theta_{i}^{allowed}$ in Equations (3) and (4) with $R_{j}$ and $\theta_{j}^{allowed}$.

(2) Task Execution: The execution time when task *i* is executed on a node with computing resources *f* is:

(5) $$t^{exe}\left(f\right)=\frac{L_{i}C_{i}}{f}$$

The execution cost [14] is:

(6) $$M^{exe}\left(f\right)=\gamma k_{c}f^{2}L_{i}C_{i}$$

When the task is executed on the server, $f=f_{i}^{server}$; when executed by assisting vehicle *j*, $f=f_{i}^{j}$. The quadratic dependence on *f* in Equation (6) follows from the widely adopted DVFS energy model [14,16], where processor power scales as $f^{3}$ and thus total energy for a fixed workload $L_{i}C_{i}$ scales as $f^{2}L_{i}C_{i}$.

(3) Cost Summary: The unit resource price of the server is $p_{i}$, and the computing service fee paid by user vehicles to the server is $P_{i}=p_{i}f_{i}^{server}$. The total expenditure of user vehicles is the sum of transmission costs and service fees:

(7) $$M_{i}=M_{i,server}^{tran}+P_{i}$$

Since assisting vehicles are selfish and will not provide services without compensation, the server needs to pay them remuneration P(i,j) (the specific determination method is described in Section 5). The server’s total expenditure for outsourced tasks is:

(8) $$M_{i,server}^{outsource}=M_{(i,server),j}^{tran}+P\left(i,j\right)$$

### 3.4. Utility Functions

(1) User Vehicle Utility: Unlike other articles that merely rely on weight factors of time and cost to reflect user utility [14,18], we adopt an exponential decay function $V_{i}\cdot e^{-\lambda_{i}\cdot \left(t_{i}/t_{i}^{\max}\right)}$ to model the realized task value under time sensitivity, where the decay factor $\lambda_{i}$ controls urgency—a larger $\lambda_{i}$ means the value drops more sharply with delay. The user utility is defined as:

(9) $$U_{i}=V_{i}\cdot e^{-\lambda_{i}\cdot \left(\frac{t_{i}}{t_{i}^{\max}}\right)}-M_{i}$$

where the first term is the realized task value, and $M_{i}$ is the total expenditure, including transmission costs and service fees. Thus, $U_{i}$ represents the net benefit: realized value minus total cost.

(2) Server Utility: The server mainly earns profits from executing user-submitted tasks and outsourcing overloaded tasks. The server utility is given below, where the first term represents the revenue from executing local tasks, and the second term represents the revenue from outsourcing some tasks. Here, $x_{i},y_{i},z_{i,j}$ take values of 0 or 1. When $x_{i}=1$, the task is arranged for execution. When $y_{i}=1$, the task is arranged for server execution. When $z_{i,j}=1$, task *i* is processed by assisting vehicle *j*. Following the settings in [4,14], each assisting vehicle accepts at most 1 task, i.e., $\sum_{i=1}^{I}z_{i,j}\leq 1$. Meanwhile, each task is assigned to at most one assisting vehicle, $\sum_{j=1}^{J}z_{i,j}\leq 1$.

(10) $$U_{server}=\sum_{i=1}^{I}x_{i}y_{i}\left[P_{i}-\gamma k_{c}{\left(f_{i}^{server}\right)}^{2}C_{i}L_{i}\right]-\sum_{i=1}^{I}\sum_{j=1}^{J}z_{i,j}\left(M_{(i,server),j}^{tran}+P\left(i,j\right)\right)$$

(3) Assisting Vehicle Utility: The revenue of assisting vehicles comes from accepting and executing tasks outsourced by the server.

(11) $$U_{j}=\sum_{i=1}^{I}z_{i,j}\cdot \left[P\left(i,j\right)-\gamma k_{c}{\left(f_{i}^{j}\right)}^{2}C_{i}L_{i}\right]$$

In summary, the three utility functions share a consistent economic structure: each entity’s utility equals its revenue minus its cost. For the server (Equation (10)), revenue comes from user service fees $P_{i}$, and costs include local execution energy and outsourcing payments. For assisting vehicles (Equation (11)), revenue comes from the server’s outsourcing payment P(i,j), and cost is the energy consumed for task execution. This differentiated utility design reflects each entity’s distinct role in the offloading ecosystem and ensures that the subsequent pricing mechanism can properly balance their respective interests.

### 3.5. Problem Formulation

In VEC task processing scenarios, task vehicles, servers, and assisting vehicles all possess rational and selfish attributes, with the core objective of maximizing their own utility. If the server prices are too high (exceeding the payment willingness threshold of task vehicles), task vehicles will choose local execution or abandon tasks; conversely, if priced too low, server profits may be impaired. During server peak load periods, the server earns margin profits by outsourcing overloaded tasks to assisting vehicles, which is the difference between user payment prices and outsourcing costs (including transmission overhead and payments to assisting vehicles). However, if the margin profit is negative (i.e., user payment price is lower than total outsourcing cost), the server will forgo outsourcing; meanwhile, if the remuneration is lower than the minimum cost for assisting vehicles to execute tasks, their participation willingness will significantly decrease. Therefore, based on in-depth analysis of the multi-agent interest game relationships in this scenario, we first establish a social welfare maximization problem to achieve the highest overall system efficiency, then apply bargaining game theory for resource pricing negotiation to resolve interest conflicts among entities. Here, “social welfare” is a standard metric in game theory and edge computing research [4,34,48], defined as the aggregate realized task value minus total system costs. First, we define the task-level social welfare, i.e., the net value generated by processing a single task *i*, which can be expressed as:

(12) $$\begin{array}{cc} W_{i}= & V_{i}\cdot e^{-\lambda_{i}\cdot \left(\frac{t_{i}}{t_{i}^{\max}}\right)}-\gamma \left[\theta_{i}^{allowed}\frac{L_{i}}{R_{i}}+\sum_{j}z_{i,j}\theta_{j}^{allowed}\frac{L_{i}}{R_{j}}\right]\\ \; & -\gamma k_{c}\left[y_{i}{\left(f_{i}^{server}\right)}^{2}+\sum_{j}z_{i,j}{\left(f_{i}^{j}\right)}^{2}\right]L_{i}C_{i} \end{array}$$

It is worth noting that since task *i* may be executed on the server or on some assisting vehicle, the transmission time and execution consumption in the task-level social welfare depend on the task’s execution location. The system-level social welfare is then defined as the sum of all task-level values $\sum_{i}x_{i}W_{i}$, representing the overall efficiency of the entire offloading system. The social welfare maximization problem can be formulated as:

(13) $$\begin{array}{cc} P1:\max\; & U^{social}\left(X,Y,Z,F^{allocated}\right)=\sum_{i=1}^{I}x_{i}W_{i}\\ s.t.\; & \left(a\right):x_{i}\in \left\{0,1\right\}\\ \; & \left(b\right):y_{i}\in \left\{0,1\right\}\\ \; & \left(c\right):y_{i}+\sum_{j}^{J}z_{i,j}\leq 1\\ \; & \left(d\right):\sum_{i}^{I}z_{i,j}\leq 1\\ \; & \left(e\right):\sum_{i}^{I}x_{i}y_{i}f_{i}^{server}\leq F_{server}\\ \; & \left(f\right):\sum_{i}^{I}x_{i}z_{i,j}f_{i}^{j}\leq f_{j}\\ \; & \left(g\right):t_{i}\leq t_{i}^{\max}\\ \; & \left(h\right):W_{i}\geq 0 \end{array}$$

In the constraints of problem **P1**: constraint (a) indicates whether the task is selected for execution; constraint (b) indicates whether the task is executed on the server; constraint (c) states that if a single task is executed, it can only be on the server or one assisting vehicle, without repeated execution; constraint (d) states that each assisting vehicle can accept at most one task; constraint (e) ensures the total computing resources required by tasks executed on the server do not exceed server computing resource capacity; constraint (f) ensures the computing resources required by tasks on an assisting vehicle do not exceed that vehicle’s available computing resource capacity; constraint (g) ensures the task execution time does not exceed the maximum tolerable time; constraint (h) ensures the task-level social welfare is non-negative.

## 4. Algorithm Design

### 4.1. DRL Problem Transformation and Social Welfare Maximization

We first solve the aforementioned mixed-integer programming problem using deep reinforcement learning methods. Under the deep reinforcement learning framework, the agent continuously optimizes its policy representation through high-frequency closed-loop interaction with the environment, achieving adaptive dynamic evolution. Its decision mechanism strictly follows the formal description of Markov Decision Processes, which can be fully characterized by the quadruple $\left(S,A,R^{\prime },P\right)$: where *S* represents the complete enumeration of the potential state space; *A* corresponds to the structured set of feasible action space; $R^{\prime }$ is the immediate reward function, quantifying the instantaneous utility of state-action-successor state; *P* is the state transition kernel, precisely characterizing the evolution probability measure of the system state under specific state–action pairs. PPO is an actor–critic deep reinforcement learning algorithm proposed by OpenAI in 2017 [49]. This algorithm introduces explicit proximal constraints in the policy gradient framework, constructs a clipped surrogate objective to implicitly limit the KL divergence between successive policy updates, thereby effectively suppressing training instability caused by excessive policy updates while ensuring sample efficiency. PPO theoretically inherits the monotonic improvement property of TRPO, and in practice combines the simplicity and scalability of REINFORCE-family algorithms, having been widely applied to continuous control, discrete decision-making, and high-dimensional policy search tasks, and is regarded as a representative method in the field.

(1) State Space: The core of edge computing is processing data on edge nodes close to data sources, with key features including resources, task heterogeneity, and communication conditions. Therefore, the state space of edge computing scenarios needs to abstract key environmental features that influence agent decisions around these characteristics. In our proposed model, we incorporate task attributes, channel states, server available resources, and assisting vehicle resource states into the state definition.

(14) $$s_{l}=\left\{T_{I},BM,F_{S},F_{J}\right\}$$

At a certain moment, state $T_{I}$ represents the task set, BM represents the communication status of all vehicles, $F_{S}$ represents the available resources of the server, and $F_{J}$ represents the available resource status of the assisting vehicle set.

(2) Action Space: To effectively solve problem **P1**, this paper designs an action space suitable for the PPO algorithm. The agent needs to make two decisions for each task: (1) select the location for processing the task, (2) determine the computing resource amount for the task. First, the agent selects a task *i* from the task set I={1,2,…,I} for processing, and after each selection, the task is removed from the pending set to ensure no repeated processing. Then, for the selected task *i*, the agent needs to decide its execution location $A_{discrete}$, with value space {0,1,2,…,J+1}. Here, 0 means abandoning execution of the task; 1 means the task is executed on the server; 2 to J+2 means assigning the task to the corresponding assisting vehicle j∈{1,2,…,J}, and each discrete variable corresponds to a continuous parameter representing its required computing resource amount $f_{i}$. Therefore, the complete action space is defined as:

(15) $$A^{c}=\underset{a\in A_{discrete}}{⋃}\left\{\left(a,z\right)|z\in R\right\}$$

where *a* represents the discrete action, and *z* is the corresponding continuous parameter. To reduce action space complexity and ensure decision effectiveness, actions are masked based on the constraints of problem **P1** before each decision. For example, through the structural design of the action space, constraints (a), (b), (c) are naturally satisfied. Constraints (d) to (h) are implemented through the action masking mechanism: before each decision, the system evaluates the feasibility of all possible actions, masks actions that violate constraints, ensuring that agents only select from the valid action set, thereby improving training efficiency and ensuring solution feasibility.

(3) Reward Function: Reward is the feedback received by the agent after executing an action during environment exploration. The objective of this paper is to evaluate the maximized social welfare after task allocation. Therefore, we define the reward obtained by the agent after each action execution as the task-level reward function basis. The agent achieves maximum social welfare by adjusting task execution locations and required resource amounts while perceiving the overall environment state:

(16) $$r_{l}=W_{i}$$

Through the above definition, we transform the original mixed-integer programming problem **P1** into an equivalent sequential decision problem **P2**. The core idea of this transformation is to reconstruct the static problem of simultaneously optimizing all decision variables into a dynamic process of making decisions step by step.

**Definition** **1.** *(Sequential Decision Problem). Given task set I={1,2,…,|I|}, define the independent decision problem **P2** as:*

(17) $$P2:\max\sum_{i=1}^{\left|I\right|}W_{i}$$

*where $W_{i}$ is the social welfare of task i, determined by execution location $p_{i}\in \left\{0,1,\ldots ,J+1\right\}$ and resource allocation amount $f_{i}$, including three cases: if $p_{i}=0$: abandon task, $W_{i}=0$; if $p_{i}=1$: server execution, resource amount $f_{i}=f_{i}^{server}$; if $p_{i}=j+1$ (j∈J): assisting vehicle j execution, resource amount $f_{i}=f_{i}^{j}$.*

**Theorem** **1.** *(Equivalence). Sequential decision problem **P2** is equivalent to original problem **P1**, i.e.,:*

(18) $$\max_{P2}\sum_{t=1}^{I}r_{t}=\max_{P1}\sum_{i=1}^{I}x_{i}W_{i}$$

*where I[·] is the indicator function, taking value 1 when the condition inside the brackets holds, and 0 otherwise. Specifically, $x_{i_{t}}=I\left[p_{t}\neq 0\right]$ indicates that if the agent does not choose to abandon at step t (i.e., $p_{t}\neq 0$), the task is marked for execution, corresponding to $x_{i}=1$ in the original problem; $y_{i_{t}}=I\left[p_{t}=1\right]$ indicates that if the agent chooses to offload the task to the server (i.e., $p_{t}=1$), it corresponds to $y_{i}=1$ in the original problem; $z_{i_{t},j}=I\left[p_{t}=j+1\right]$ (j∈{1,…,J}) indicates that if the agent chooses to assign the task to assisting vehicle j (i.e., $p_{t}=j+1$), it corresponds to $z_{i,j}=1$ in the original problem. Since each task is selected exactly once in the sequential decision process, and the execution location at each decision step is mutually exclusive (only one of abandonment, server, or a specific assisting vehicle is chosen), the above indicator functions naturally satisfy the integrality and mutual exclusivity requirements of constraints (a)–(d) in the original problem **P1**. Combined with constraints (e)–(h) being guaranteed through feasibility checking at each decision step, the optimal values of the two problems are the same.*

This sequential transformation decomposes the multi-dimensional joint decision space into multiple steps of low-dimensional subspaces, where each step only needs to consider remaining tasks and allocated resources, avoiding global search. Based on this transformation, the sequential decision structure perfectly fits the MDP framework, and the PPO algorithm learns policy $\pi_{\theta }\left(a|s\right)$ to maximize cumulative reward $E[\sum_{t=1}^{I}r_{t}]$, thereby solving the original optimization problem **P1**.

### 4.2. Policy Learning Based on Hybrid Action Representation

**Figure 3:**
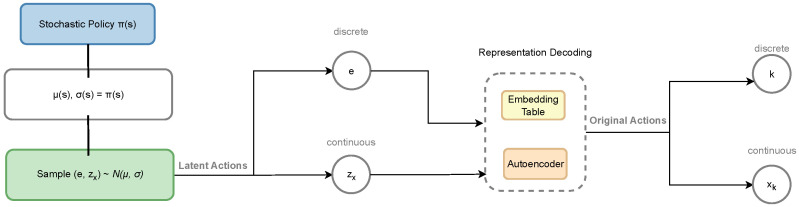
HyAR-PPO algorithm architecture.

In the previous section, we modeled the VEC task offloading problem as a hybrid action space reinforcement learning problem, where discrete actions represent task execution locations and continuous parameters represent resource allocation amounts. However, traditional reinforcement learning algorithms have difficulty directly handling such heterogeneous hybrid action spaces. This section details the core principles of the Hybrid Action Representation (HyAR) framework and proposes the HyAR-PPO algorithm, combining HyAR with PPO. To our knowledge, this is the first time the HyAR framework has been applied to the VEC domain and combined with the stochastic policy algorithm PPO to achieve policy optimization in hybrid action spaces. The architecture of the proposed HyAR-PPO algorithm is shown in Figure 3.

The core idea of HyAR [47] is to construct a unified, decodable latent representation space that maps heterogeneous discrete–continuous hybrid actions to a homogeneous continuous latent space, enabling traditional continuous control algorithms to be applied. The HyAR framework consists of three core components: discrete action embedding table, conditional variational autoencoder, and environment dynamics predictor.

(1) Discrete Action Embedding Table: For discrete action set K={1,2,…,K}, HyAR maintains a learnable embedding table $E_{\zeta }\in R^{K\times d_{1}}$, where each row $e_{\zeta ,k}=E_{\zeta }\left(k\right)$ is the $d_{1}$-dimensional continuous vector representation of discrete action *k*. This embedding mechanism transforms discrete action selection into a vector lookup problem in continuous space.

(2) Conditional Variational Autoencoder (Conditional VAE): For continuous parameter $x_{k}$, HyAR uses a conditional VAE to construct its latent representation space. Given state *s*, discrete action *k* and its embedding $e_{\zeta ,k}$, the encoder $q_{\phi }\left(z|x_{k},s,e_{\zeta ,k}\right)$ maps continuous parameter $x_{k}$ to latent variable $z\in R^{d_{2}}$; the decoder $p_{\psi }\left({\tilde{x}}_{k}|z,s,e_{\zeta ,k}\right)$ reconstructs continuous parameters from latent variable *z*. The encoder outputs Gaussian distribution $N(\mu_{x},\sigma_{x})$, and sampling is performed through the reparameterization trick $z=\mu_{x}+\sigma_{x}⊙\epsilon$ (where ϵ∼N(0,I)), enabling gradients to backpropagate.

(3) Environment Dynamics Prediction: To make the latent representation space semantically smooth (i.e., similar latent representations correspond to similar environmental impacts), HyAR introduces environment dynamics prediction as an auxiliary task. For state transition $\left(s,k,x_{k},s^{\prime }\right)$, the predictor outputs state residual prediction ${\tilde{\delta }}_{s,s^{\prime }}=p_{\psi }\left(z_{x},s,e_{\zeta ,k}\right)$, where the true state residual $\delta_{s,s^{\prime }}=s^{\prime }-s$. This design ensures that similar action representations in the latent space correspond to similar environmental dynamics effects.

The training loss function of the hybrid action representation model consists of VAE reconstruction loss and dynamics prediction loss:

(19) $$L_{VAE}\left(\phi ,\psi ,\zeta \right)=E_{s,k,x_{k}∼D,z∼q_{\phi }}\left[∥x_{k}-{\tilde{x}}_{k}{∥}_{2}^{2}+D_{KL}\left(q_{\phi }\left(\cdot |x_{k},s,e_{\zeta ,k}\right)∥N\left(0,I\right)\right)\right]$$

where the first term is the reconstruction error and the second term is the KL divergence regularization term, ensuring the latent variable distribution is close to a standard Gaussian distribution.

The dynamics prediction loss is defined as:

(20) $$L_{Dyn}\left(\phi ,\psi ,\zeta \right)=E_{s,k,x_{k},s^{\prime }}\left[∥{\tilde{\delta }}_{s,s^{\prime }}-\delta_{s,s^{\prime }}{∥}_{2}^{2}\right]$$

The total loss function of the hybrid action representation model is:

(21) $$L_{HyAR}\left(\phi ,\psi ,\zeta \right)=L_{VAE}\left(\phi ,\psi ,\zeta \right)+\beta L_{Dyn}\left(\phi ,\psi ,\zeta \right)$$

where β is the weight coefficient of the dynamics prediction loss.

**Figure 4:**
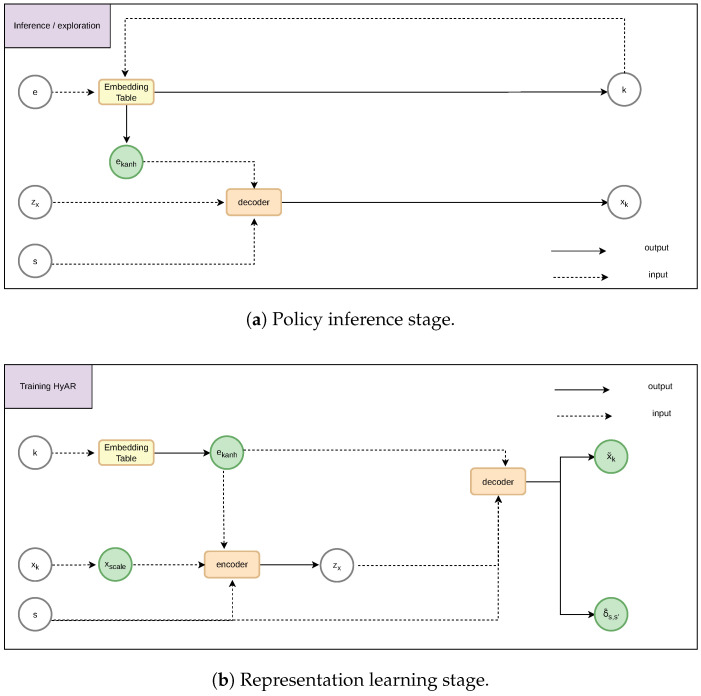
HyAR working mechanism.

The encoding and decoding process of hybrid actions can be formalized as:

(22) $$\mathrm{Encoding}:\;e_{\zeta ,k}=E_{\zeta }\left(k\right),\;z_{x}∼q_{\phi }\left(\cdot |x_{k},s,e_{\zeta ,k}\right)$$

(23) $$\mathrm{Decoding}:\;k=g_{E}\left(e\right)=\arg\min_{k^{\prime }\in K}{∥e_{\zeta ,k^{\prime }}-e∥}_{2},\;x_{k}=p_{\psi }\left(z_{x},s,e_{\zeta ,k}\right)$$

where discrete action decoding uses nearest neighbor lookup, finding the discrete action closest to the latent vector *e* in the embedding table. In implementation, following [47], both the discrete action embedding and continuous parameters are scaled to the range [−1,1] before being fed into the network: the embedding vector is normalized via a tanh activation to produce $e_{k}^{tanh}=\tanh\left(e_{\zeta ,k}\right)$, and the continuous parameter is linearly rescaled to $x_{scale}\in \left[-1,1\right]$. This normalization ensures that all latent dimensions share a consistent scale, which stabilizes training and improves representation quality. These intermediate variables $e_{k}^{tanh}$ and $x_{scale}$ are shown in Figure 4. As shown in Figure 4, the policy inference stage does not use the Encoder. The policy network directly outputs latent actions $\left(e,z_{x}\right)$, the discrete action *k* is obtained through reverse lookup in the Embedding Table, and the Decoder decodes to obtain continuous parameters $x_{k}$. The representation learning stage uses the Encoder to encode original actions $\left(k,x_{k}\right)$ and state *s* into latent variable $z_{x}$, and the Decoder is responsible for reconstructing continuous parameters ${\tilde{x}}_{k}$ and predicting state changes ${\tilde{\delta }}_{s,s^{\prime }}$.

Based on the above HyAR framework, this paper adopts PPO as the base reinforcement learning algorithm to construct HyAR-PPO. PPO uses a stochastic policy, where the policy network outputs action distribution parameters rather than deterministic action values. In HyAR-PPO, the latent policy network $\pi_{\omega }$ outputs Gaussian distribution parameters for discrete action embedding *e* and continuous action latent variable $z_{x}$, respectively:

(24) $$e∼N\left(\mu_{e}\left(s\right),\sigma_{e}\left(s\right)\right),\;z_{x}∼N\left(\mu_{z}\left(s\right),\sigma_{z}\left(s\right)\right)$$

where $\mu_{e}\left(s\right)\in R^{d_{1}}$, $\sigma_{e}\left(s\right)\in R^{d_{1}}$ are the mean and standard deviation vectors of discrete action embedding; $\mu_{z}\left(s\right)\in R^{d_{2}}$, $\sigma_{z}\left(s\right)\in R^{d_{2}}$ are the mean and standard deviation vectors of the continuous action latent variable. The two distributions are independent and are sampled and optimized separately. Stochastic policies naturally explore through the variance of action distributions without additional noise, and the degree of exploration is learnable; meanwhile, PPO uses on-policy learning, where each update uses data collected by the current policy, avoiding distribution shift problems.

The core of PPO is the Clipped Surrogate Objective, which limits the update magnitude between old and new policies to ensure training stability:

(25) $$L^{CLIP}\left(\omega \right)=E_{t}\left[\min\left(r_{t}\left(\omega \right){\hat{A}}_{t},\mathrm{clip}\left(r_{t}\left(\omega \right),1-\epsilon ,1+\epsilon \right){\hat{A}}_{t}\right)\right]$$

where the importance sampling ratio is defined as:

(26) $$r_{t}\left(\omega \right)=\frac{\pi_{\omega }\left(a_{t}|s_{t}\right)}{\pi_{\omega_{old}}\left(a_{t}|s_{t}\right)}$$

${\hat{A}}_{t}$ is the advantage function estimate, and ϵ is the clipping parameter. The clipping operation ensures that when $r_{t}\left(\omega \right)$ deviates too far from 1, the gradient is clipped, thereby limiting the policy update magnitude.

Since *e* and $z_{x}$ use independent Gaussian distributions, HyAR-PPO calculates two importance sampling ratios separately:

(27) $$r_{t}^{e}\left(\omega \right)=\frac{N(e_{t}|\mu_{e}\left(s_{t}\right),\sigma_{e}\left(s_{t}\right))}{N(e_{t}|\mu_{e,old}\left(s_{t}\right),\sigma_{e,old}\left(s_{t}\right))},\;r_{t}^{z}\left(\omega \right)=\frac{N(z_{x,t}|\mu_{z}\left(s_{t}\right),\sigma_{z}\left(s_{t}\right))}{N(z_{x,t}|\mu_{z,old}\left(s_{t}\right),\sigma_{z,old}\left(s_{t}\right))}$$

Correspondingly, the policy loss is the sum of two parts: $L^{CLIP}=L_{e}^{CLIP}+L_{z}^{CLIP}$, performing clipped optimization on the distributions of *e* and $z_{x}$, respectively.

The advantage function is calculated using Generalized Advantage Estimation (GAE) to balance bias and variance:

(28) $${\hat{A}}_{t}=\sum_{l=0}^{\infty }{\left(\gamma \lambda \right)}^{l}\delta_{t+l},\;\delta_{t}=r_{t}+\gamma V_{\theta }\left(s_{t+1}\right)-V_{\theta }\left(s_{t}\right)$$

where γ is the discount factor, λ is the GAE parameter, and $V_{\theta }$ is the state value function network.

HyAR-PPO adopts a two-stage training strategy. Stage 1 (Warmup Stage): Use a random policy to interact with the environment and collect data, train the hybrid action representation model (embedding table $E_{\zeta }$ and conditional VAE $q_{\phi },p_{\psi }$), and establish a meaningful latent representation space. In this stage, the policy network is not updated, only action representation is learned by minimizing $L_{HyAR}$. Following [47], the warmup ratio is set to 5% of total training episodes. Stage 2 (Training Stage): Train the PPO policy network in the learned latent representation space. At the same time, continue to update the representation model at a lower frequency to adapt to changes in data distribution. Algorithm 1 provides the complete pseudocode of HyAR-PPO.
**Algorithm 1** HyAR-PPO Algorithm**Require:** Warmup episodes $N_{warmup}$, training episodes $N_{train}$, representation update interval $N_{rep}$
**Ensure:** Trained latent policy network $\pi_{\omega }^{*}$
  1: Initialize: latent policy network $\pi_{\omega }$, value network $V_{\theta }$, embedding table $E_{\zeta }$, conditional VAE $q_{\phi },p_{\psi }$
  2: **// Stage 1: Warmup Training (Learn Action Representation)**
  3: **for**
episode=1 to $N_{warmup}$ **do**
  4:     Collect data $\left\{s,k,x_{k},s^{\prime }\right\}$ with random policy, encode to get $\left\{e_{\zeta ,k},z_{x}\right\}$ {Equation (22)}
  5:     Minimize $L_{HyAR}$ to update $E_{\zeta },q_{\phi },p_{\psi }$ {Equation (21)}
  6: **end for**
  7: **// Stage 2: Policy Training (Latent Space Policy Optimization)**
  8: **for**
episode=1 to $N_{train}$ **do**
  9:     **for** t=0 to T−1 **do**
10:        Sample $e_{t}∼N\left(\mu_{e}\left(s_{t}\right),\sigma_{e}\left(s_{t}\right)\right)$, $z_{x,t}∼N\left(\mu_{z}\left(s_{t}\right),\sigma_{z}\left(s_{t}\right)\right)$ {Equation (24)}
11:        Decode: $k_{t}=g_{E}\left(e_{t}\right)$, $x_{k,t}=p_{\psi }\left(z_{x,t},s_{t},e_{\zeta ,k_{t}}\right)$ {Equation (23)}
12:        Execute $\left(k_{t},x_{k,t}\right)$, observe $r_{t},s_{t+1}$
13:    **end for**
14:    Compute GAE advantage estimate ${\hat{A}}_{t}$ {Equation (28)}
15:    **for** epoch=1 to *K* **do**
16:        Maximize $L^{CLIP}$ to update $\pi_{\omega }$ {Equation (25)}
17:        Update state value function network $V_{\theta }$
18:    **end for**
19:    **if** $episode\;\mathrm{mod}\;N_{rep}=0$ **then**
20:        Minimize $L_{HyAR}$ to update representation model {Equation (21)}
21:     **end if**
22: **end for**
23: **return** $\pi_{\omega }^{*}$


## 5. Pricing Strategy

In the bargaining game of this scenario, users submit task offloading requests as buyers purchasing computing resources, and the server executes tasks as a seller of computing resources. At the same time, due to the insufficient single-point computing resources of the server, the server also acts as a buyer, paying certain incentive fees to use assisting vehicles that provide computing resources. In a bargaining game, participants mutually game over resource pricing, and the Nash bargaining solution assigns an equilibrium outcome to the game. This outcome can be an agreement point or a disagreement point. The Nash bargaining solution of each game is regarded as the negotiation result of all parties, i.e., no party has the motivation to unilaterally change the outcome.

In the case of reaching an agreement, users pay reasonable fees to obtain computing resources, the server allocates computing resources and executes tasks, and the product of all parties’ utilities is maximized. In the case of disagreement, it indicates that pricing has harmed some party’s utility and cannot satisfy all parties, so negotiation fails. Since Algorithm 1 reasonably allocates all tasks, the interests of participants can be guaranteed.

When a task is executed on the server, a bilateral game between the user and server is formed. According to Nash bargaining theory, combined with Equations (9) and (10) describing both parties’ utilities, the equilibrium price is solved by maximizing the Nash product:

(29) $$\max_{p_{i}}\Pi ={\left(U_{i}-d_{i}\right)}^{\alpha_{1}}\cdot {\left(U_{server}^{i}-d_{server}\right)}^{1-\alpha_{1}}$$

where $d_{i}=0$ and $d_{server}=0$ are the disagreement point utilities of both parties (i.e., utilities when no agreement is reached); $\alpha_{1}\in \left[0,1\right]$ represents the relative bargaining power of the user, reflecting the user’s position strength in negotiation; $\left(1-\alpha_{1}\right)$ correspondingly represents the relative bargaining power of the server. Solving through the first-order condition $\frac{\partial \Pi }{\partial p_{i}}=0$, we obtain the equilibrium price:

(30) $$p_{i}^{*}=\alpha_{1}\cdot p_{min}+\left(1-\alpha_{1}\right)\cdot p_{max}$$

where $p_{min}$ is the server’s reservation price (covering execution costs), and $p_{max}$ is the user’s maximum willingness to pay (based on task value minus transmission costs). This linear combination reflects the weighted influence of both parties’ bargaining power on the final price.

When a task is assigned to an assisting vehicle, a three-party game among the users, the server, and the assisting vehicle is formed. According to the utilities defined in Equations (9)–(11), the three-party Nash bargaining problem is:

(31) $$\max_{P_{i},P\left(i,j\right)}\Pi ={\left(U_{i}\right)}^{\alpha_{1}}\cdot {\left(U_{server}^{i}\right)}^{\alpha_{2}}\cdot {\left(U_{j}\right)}^{\alpha_{3}}$$

Constraint: $\alpha_{1}+\alpha_{2}+\alpha_{3}=1$. For simplification, let $A_{i}=V_{i}-M_{i}^{trans}$, $B=M_{(i,server),j}^{trans}$, $C_{j}=M_{j}^{exe}$, then the utilities of all parties can be expressed as $U_{i}=A_{i}-P_{i}$, $U_{server}^{i}=P_{i}-B-P\left(i,j\right)$, $U_{j}=P\left(i,j\right)-C_{j}$.

Taking the logarithm and deriving first-order conditions:

(32) $$\frac{\partial \ln\Pi }{\partial P_{i}}=-\frac{\alpha_{1}}{A_{i}-P_{i}}+\frac{\alpha_{2}}{P_{i}-B-P\left(i,j\right)}=0$$

(33) $$\frac{\partial \ln\Pi }{\partial P(i,j)}=-\frac{\alpha_{2}}{P_{i}-B-P\left(i,j\right)}+\frac{\alpha_{3}}{P\left(i,j\right)-C_{j}}=0$$

Define cooperation surplus $K=A_{i}-B-C_{j}$, i.e., the sum of three-party utilities. Since the action masking mechanism of Algorithm 1 ensures K>0, solving the above equation system yields equilibrium prices:

(34) $$P_{i}^{*}=A_{i}-\alpha_{1}K=\left(V_{i}-M_{i}^{trans}\right)-\alpha_{1}K$$

(35) $$P{\left(i,j\right)}^{*}=C_{j}+\alpha_{3}K=M_{j}^{exe}+\alpha_{3}K$$

This solution shows that the utility obtained by each party equals exactly their bargaining power ratio times the total cooperation surplus, achieving Pareto-optimal surplus allocation. Finally, we summarize the entire system’s business process as shown in Algorithm 2.
**Algorithm 2** Incentive-Driven Utility-Balanced Task Offloading Framework**Require:** Task set I, assisting vehicle set J, server resources *F*
**Ensure:** Allocation scheme (X,Y,Z), equilibrium prices
  1: **// Task Allocation**
  2: **for** each task i∈I **do**
  3:     Use $\pi_{\omega }^{*}$ to determine offloading location and resource allocation
  4: **end for**
  5: **// Equilibrium Pricing**
  6: **for** each allocated task *i* **do**
  7:     **if** $y_{i}=1$ (server execution) **then**
  8:         Bilateral game pricing {Equation (30)}
  9:     **else if** $z_{i,j}=1$ (assisting vehicle execution) **then**
10:         Three-party game pricing {Equations (34) and (35)}
11:     **end if**
12: **end for**
13: **return** Allocation scheme and equilibrium prices


### Analysis of Pricing Mechanism Properties

This section theoretically verifies that the designed Nash bargaining pricing mechanism satisfies Individual Rationality (IR) constraints and achieves Pareto-optimal and fair cooperation surplus allocation.

**Definition** **2.** *(Individual Rationality). If a mechanism satisfies $U_{i}\geq d_{i}$ for all participants i, where $d_{i}$ is participant i’s reservation utility (disagreement point utility), then the mechanism is said to satisfy the individual rationality constraint.*

In our scenario, the reservation utility of all parties is 0, i.e., $d_{i}=d_{server}=d_{j}=0$. We prove the satisfaction of IR constraints in bilateral and three-party game scenarios below.

**Proposition** **1.** *(Bilateral Game IR). In the user–server bilateral game, the equilibrium price $p_{i}^{*}=\alpha_{1}\cdot p_{min}+\left(1-\alpha_{1}\right)\cdot p_{max}$ satisfies the IR constraint.*

**Proof.**  By the definition of equilibrium price, $p_{min}$ is the server’s reservation price (covering execution costs), and $p_{max}$ is the user’s maximum willingness to pay. Since $\alpha_{1}\in \left[0,1\right]$, the equilibrium price satisfies $p_{min}\leq p_{i}^{*}\leq p_{max}$. Therefore, user utility $U_{i}=V_{i}\cdot e^{-\lambda_{i}\cdot \left(t_{i}/t_{i}^{\max}\right)}-M_{i}^{trans}-p_{i}^{*}f_{i}^{server}\geq 0$; server utility $U_{server}^{i}=p_{i}^{*}f_{i}^{server}-M_{server}^{exe}\geq 0$. Hence, the bilateral game satisfies the IR constraint. □

**Proposition** **2.** *(Three-Party Game IR). In the user–server-assisting vehicle three-party game, the equilibrium prices $P_{i}^{*}=A_{i}-\alpha_{1}K$ and $P{\left(i,j\right)}^{*}=C_{j}+\alpha_{3}K$ satisfy the IR constraint.*

**Proof.**  Let $A_{i}=V_{i}-M_{i}^{trans}$, $B=M_{(i,server),j}^{trans}$, $C_{j}=M_{j}^{exe}$, cooperation surplus $K=A_{i}-B-C_{j}$. By the action masking mechanism of Algorithm 1, tasks are assigned to assisting vehicles only when K>0, i.e., cooperation surplus is positive. The utilities of all parties are: user utility $U_{i}=A_{i}-P_{i}^{*}=\alpha_{1}K>0$; server utility $U_{server}^{i}=P_{i}^{*}-B-P{\left(i,j\right)}^{*}=\alpha_{2}K>0$; assisting vehicle utility $U_{j}=P{\left(i,j\right)}^{*}-C_{j}=\alpha_{3}K>0$. Since K>0 and $\alpha_{1},\alpha_{2},\alpha_{3}>0$, all parties’ utilities are strictly greater than 0, satisfying the IR constraint. □

**Theorem** **2.** *(Mechanism Properties). The Nash bargaining pricing mechanism designed in this paper has the following properties:*

*1.*  *Individual Rationality: All participants’ utilities are no less than their reservation utilities, ensuring that all parties have motivation to participate in cooperation.*
*2.*  *Pareto Optimality: Cooperation surplus is fully allocated, i.e., $U_{i}+U_{server}^{i}+U_{j}=K$, and there is no reallocation scheme that increases some party’s utility without harming others.*
*3.*  *Fairness: Each party’s obtained utility equals exactly their bargaining power ratio times the total cooperation surplus, i.e., $U_{i}=\alpha_{1}K$, $U_{server}^{i}=\alpha_{2}K$, $U_{j}=\alpha_{3}K$, reflecting the symmetry of the Nash bargaining solution.*

The above properties are guaranteed by the four core axioms of Nash bargaining theory: Pareto efficiency, symmetry, affine transformation invariance, and independence of irrelevant alternatives [48].

## 6. Experiments

### 6.1. Experimental Environment and Parameter Settings

All simulation experiments in this paper are executed under the Ubuntu 24.04 WSL2 environment, with hardware configuration of AMD Ryzen 5 7640HS processor with Zen 4 architecture (4.0 GHz base frequency, up to 5.0 GHz boost) and 16GB DDR5 memory. The programming language is Python 3.10, and the deep learning framework uses PyTorch 2.5.1. To ensure experimental reproducibility, all comparison methods were trained and tested under the same simulation environment and evaluation process, and results from different random seeds were averaged.

In the simulation experiments, we set the number of task vehicles in the range [5,30] and the number of assisting vehicles in the range [2,10]. Other main simulation parameters and their reference sources are shown in Table 2. These parameters together cover typical VEC scenarios from low load to high load, from scarce to abundant assisting resources, to verify the adaptability and robustness of algorithms under different system pressures.

To verify the effectiveness of the proposed method, this paper proposes the HyAR-PPO algorithm and compares it with the following methods:

- **Random**: A random baseline that uniformly samples discrete actions (offloading location) and continuous parameters (resource allocation amount), subject to feasibility constraints.
- **Server**: Server-only offloading algorithm, all tasks are executed by the edge server without using assisting vehicle resources [50].
- **HORA** [4]: Hybrid action policy method based on H-PPO parallel multi-head architecture.
- **HyAR-TD3** [47]: Hybrid action representation learning method based on TD3.

Meanwhile, to evaluate the comparison effect under two experimental settings, this paper uses two output types in plotting and statistics: The key HyAR-specific hyperparameters follow the original HyAR paper [47]: $d_{1}=d_{2}=6$, $\alpha_{recon}=2.0$, $\alpha_{KL}=0.5$, β=10.0.

- **HyAR Intra-group Comparison**: Comparing HyAR family methods (HyAR-PPO, HyAR-PPO-C, HyAR-TD3, HyAR-TD3-C), where “-C” denotes the version without warmup.
- **Baseline Comprehensive Comparison**: Comparing HyAR-PPO with main baseline methods (Random, Server, HORA, HyAR-TD3), not including “-C” control versions.

In experimental evaluation, we designed three comparison scenarios to comprehensively verify algorithm performance: (1) **Increasing User Count Scenario**: Fix the number of assisting vehicles at 10, gradually increase the number of task vehicles (5 to 30). (2) **Increasing AV Count Scenario**: Fix the number of task vehicles at 30, gradually increase the number of assisting vehicles (2 to 10). (3) **Scale Comparison**: Use typical small, medium, and large scale configurations (small: 10 tasks, 2 assistants; medium: 20 tasks, 5 assistants; large: 30 tasks, 8 assistants). These three configurations are designed to uniformly cover small, medium, and large scales within the parameter range (task vehicles [5,30], assisting vehicles [2,10]), comprehensively verifying algorithm adaptability under different system pressures. The task-to-AV ratios across the three scales are 5:1, 4:1, and 3.75:1, respectively, reflecting the practical situation that the growth of assisting resources typically lags behind the growth of task demand as the system scales up.

### 6.2. Experimental Results Analysis

#### 6.2.1. Convergence Analysis

**Figure 5:**
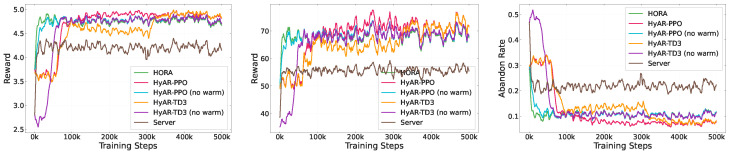
Convergence analysis of different algorithms.

To verify the convergence performance of each algorithm, we conducted training process comparisons under arbitrary scale scenarios (task vehicle number 1–30, assisting vehicle number 1–10). Figure 5 shows the training curves of each algorithm on three key metrics: average reward per task, cumulative average reward per episode, and task abandonment rate.

From Figure 5, we can observe that policies based on hybrid action representation learning are overall superior in training stability and final reward: their average reward converges faster with smaller fluctuations and can maintain task abandonment rate at a lower level. The HORA algorithm exhibits stable performance but remains at a second-tier level. The server-only offloading baseline has higher abandonment rates, indicating that introducing assisting vehicles and making effective joint decisions is necessary for improving system utility in VEC scenarios. Overall, this result shows that providing structured representations and more effective policy optimization mechanisms for hybrid action spaces helps improve training convergence and final decision quality, providing a foundation for subsequent comparative analysis.

#### 6.2.2. HyAR Intra-Group Ablation Experiment

**Figure 6:**
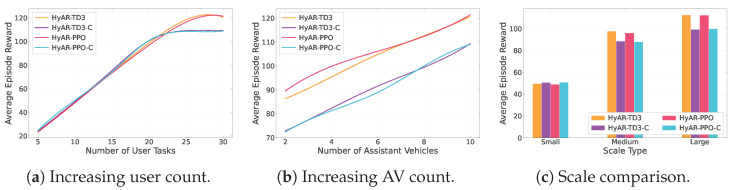
Social welfare comparison of HyAR family methods in different scenarios.

As shown in Figure 6, we systematically compared the social welfare of four HyAR family variants across three types of scenarios. In the increasing user count scenario (Figure 6a), as task vehicles increase from 5 to 30, HyAR-PPO and HyAR-TD3 with warmup maintain the highest and comparable social welfare at all load points, forming the first tier together; while the non-warmup versions HyAR-PPO-C and HyAR-TD3-C follow the same overall trend but show notable slowdown in reward growth after the task count exceeds 20, with the gap to warmup versions gradually widening. In the increasing AV count scenario (Figure 6b), both HyAR-PPO and HyAR-TD3 can continuously and fully improve rewards as assisting resources increase, while non-warmup versions show small gaps from warmup versions when assisting vehicles are few, but their reward improvement becomes limited as available resources grow, failing to fully exploit additional computing power. In the scale comparison scenario (Figure 6c), from small to large scale, warmup HyAR-PPO and HyAR-TD3 consistently maintain the lead, while non-warmup versions exhibit certain performance losses across all scales. The above results demonstrate that the structural initialization provided by the warmup stage for hybrid action representations plays a critical role in effective exploration and stable optimization of policies in high-dimensional hybrid action spaces; without warmup, policies are more prone to falling into suboptimal solutions, resulting in reward degradation.

#### 6.2.3. Comprehensive Comparison Experiment with Baseline Methods

**Figure 7:**
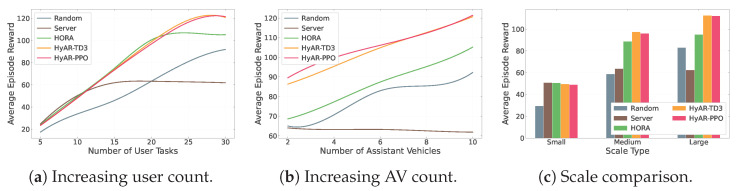
Social welfare comparison of different methods in multiple scenario types.

As shown in Figure 7, we comprehensively compared HyAR-PPO with four baseline methods across three types of scenarios.

In the increasing user count scenario (Figure 7a), as the number of task vehicles increases from 5 to 30, HyAR-PPO and HyAR-TD3 achieve the highest and comparable social welfare at all load points, jointly leading other methods. Although HORA also belongs to DRL-based hybrid action methods, its social welfare is notably lower than HyAR-PPO and HyAR-TD3, with the gap becoming more pronounced in high-load intervals. The Server baseline shows small differences from learning methods when task numbers are few, but as task numbers increase, its social welfare growth gradually plateaus due to an inability to utilize assisting vehicle resources. The Random baseline is at the lowest level across all configurations.

In the increasing AV count scenario (Figure 7b), with 30 task vehicles fixed and assisting vehicles increasing from 2 to 10, HyAR-PPO and HyAR-TD3 most effectively exploit additional assisting resources, with social welfare continuously and stably improving as assisting vehicle numbers increase. HORA also benefits from expanded assisting resources, but its improvement magnitude is notably weaker than HyAR series methods, with the gap further widening when assisting vehicles are abundant. The Server baseline exhibits a slight decline in social welfare (approximately 2–3) as assisting vehicles increase, due to degraded per-vehicle channel quality with more participants sharing the NOMA channel.

In the scale comparison scenario (Figure 7c), from small scale (10 tasks, 2 assistants) to large scale (30 tasks, 8 assistants), HyAR-PPO and HyAR-TD3 achieve optimal social welfare at medium and large scales, with HORA showing notable gaps at medium-to-large scales. It is worth noting that performance across methods is relatively close in small-scale scenarios, because the problem scale is small and constraints are relatively relaxed, allowing simple policies to achieve near-optimal solutions. Aggregating experimental data across all configurations in the three scenario types, the hybrid action representation learning-based methods (HyAR-PPO and HyAR-TD3) achieve an average social welfare improvement of approximately 11% over HORA, with more pronounced advantages in medium-to-large scale scenarios with sufficient assisting vehicles (maximum improvement reaching 30.51%). This result validates that hybrid action representation learning, by constructing compact latent representation spaces and enhancing the correlation modeling between discrete and continuous actions, can achieve superior resource allocation policies in high-dimensional hybrid decision spaces.

#### 6.2.4. Bargaining Mechanism Effect Analysis

**Figure 8:**
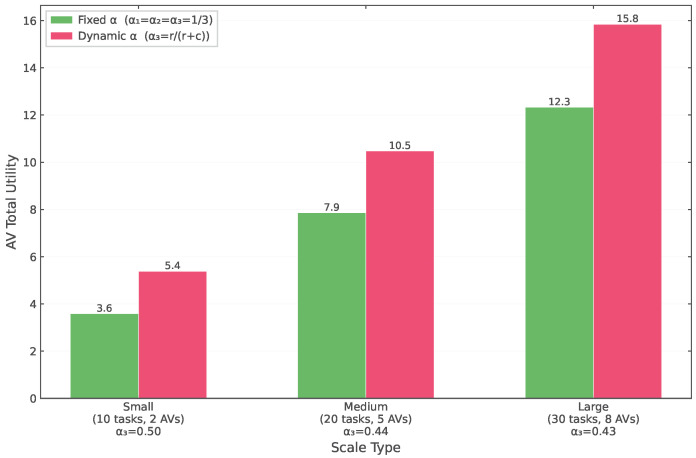
Total assisting vehicle utility comparison under different bargaining power allocation strategies.

To verify the effectiveness of the Nash bargaining pricing mechanism proposed in this paper, we compared two bargaining power allocation strategies: fixed bargaining power ($\alpha_{1}=\alpha_{2}=\alpha_{3}=1/3$) and dynamic bargaining power ($\alpha_{3}=r/\left(r+c\right)$, where *r* is the resource amount contributed by assisting vehicles and *c* is their computation cost). As shown in Figure 8, the dynamic bargaining power strategy can significantly improve the total utility of assisting vehicles across different scale scenarios.

In the small-scale scenario (10 tasks, 2 assisting vehicles), the total utility of assisting vehicles under the dynamic strategy reaches 15.8, representing a 28.5% improvement over the fixed strategy’s 12.3; in the medium-scale scenario (20 tasks, 5 assisting vehicles), the dynamic strategy utility is 10.5, representing a 32.9% improvement over the fixed strategy’s 7.9; in the large-scale scenario (30 tasks, 8 assisting vehicles), the dynamic strategy utility is 8.0, representing a 48.1% improvement over the fixed strategy’s 5.4. This indicates that dynamic bargaining power allocation based on resource contribution can more fairly reflect the actual contributions of assisting vehicles, thereby providing stronger participation incentives.

The $\alpha_{3}$ values in the dynamic strategy vary with scenario scale: 0.50 for small scale, 0.44 for medium scale, and 0.43 for large scale. This reflects that as the system scale expands, the relative bargaining power of assisting vehicles slightly decreases, but still significantly exceeds the 0.33 of the fixed strategy, embodying reasonable compensation for resource contributors.

These experimental results validate the effectiveness of the Nash bargaining pricing mechanism proposed in this paper in achieving fair profit distribution and incentivizing assisting vehicle participation, providing a theoretical foundation and practical guidance for multi-party collaboration in VEC scenarios.

## 7. Conclusions

This paper addresses the joint optimization problem of resource allocation and pricing incentives in VEC environments, proposing an incentive-driven utility-balanced task offloading framework that achieves coordinated optimization of offloading decisions, resource configuration, and profit distribution. The effectiveness of the proposed framework in the constructed scenarios has been verified through experiments. Meanwhile, this paper introduces the representation learning idea for hybrid action decisions into the VEC domain, providing methodological reference and experimental foundation for subsequent research on offloading optimization for more complex hybrid action structures.

Future work will further extend to more realistic vehicular network scenarios, such as multi-server collaboration, dynamic topologies, and incomplete information interaction, and construct higher-dimensional hybrid action settings to systematically evaluate the scalability and generalization ability of the methods. In addition, incorporating security and privacy-preserving mechanisms into the task offloading process is an important direction for practical VEC deployment.

## Figures and Tables

**Table 1:** Summary of notations.

Symbol	Description	Symbol	Description	Symbol	Description
I	Set of user vehicles	J	Set of assisting vehicles	$T_{i}$	Task generated by user *i*
$L_{i}$	Task data size	$C_{i}$	Computation complexity	$t_{i}^{\max}$	Task time tolerance
$V_{i}$	Task value assessment	$\lambda_{i}$	Value decay factor	*F*	Server computing resources
$f_{i}^{server}$	Server allocated resources	$f_{i}^{j}$	Resources provided by AV	$f_{j}$	Total AV resources
$p_{i}$	Unit resource price	$P_{i}$	Fee charged by server	P(i,j)	Outsourcing payment
γ	Unit energy price	$k_{c}$	Energy consumption coeff.	*B*	Communication bandwidth
$U_{i}$	User vehicle utility	$U_{server}$	Server utility	$U_{j}$	Assisting vehicle utility
$W_{i}$	Task-level social welfare				

**Table 2:** Main simulation parameter settings and reference sources.

Parameter	Value/Range	Parameter	Value/Range
Task data size $L_{i}$	[1,5] Mbits [18]	Complexity $C_{i}$	500 cycle/bit [18]
Delay tolerance $t_{i}^{\max}$	[5,10] s [7]	Task value $V_{i}$	[5,10] $ [16]
Decay factor $\lambda_{i}$	[0.01,0.45]	Bandwidth *B*	20 MHz [7]
Distance $dist_{k}$	[0,500] m [7]	Path loss ϕ	3.0 [7]
Noise $N_{0}$	−90 dBm [7]	Tx power $\theta_{server}$	1.0 W [7]
Server resources *F*	[6,7] GHz [4]	AV resources $f_{j}$	[1,2] GHz [4]
Energy coeff. $k_{c}$	$10^{-27}$ [16]	Energy price γ	1.0 $ [16]

## Data Availability

The original contributions presented in this study are included in the article. Further inquiries can be directed to the corresponding author.

## Abbreviations

The following abbreviations are used in this manuscript:

VEC	Vehicular Edge Computing
RSU	Roadside Unit
AV	Assisting Vehicle
UV	User Vehicle
CSP	Computation Service Provider
MINLP	Mixed-Integer Nonlinear Programming
DRL	Deep Reinforcement Learning
PPO	Proximal Policy Optimization
HyAR	Hybrid Action Representation
VAE	Variational Autoencoder
MDP	Markov Decision Process
GAE	Generalized Advantage Estimation
IR	Individual Rationality
NOMA	Non-Orthogonal Multiple Access
QoS	Quality of Service
MEC	Multi-access Edge Computing
